# Humoral Response to SARS-CoV-2 Vaccine-Boost in Cancer Patients: A Case Series from a Southern European Cancer Center

**DOI:** 10.3390/vaccines12111207

**Published:** 2024-10-24

**Authors:** Júlio Oliveira, Pedro Cruz, Tânia R. Dias, Mário Sousa-Pimenta, Beatriz Almeida, Bruno Soares, Hugo Sousa, Rui Costa, Carlos Ochoa, Francisca Dias, Rui Medeiros

**Affiliations:** 1 Department of Medical Oncology, Portuguese Institute of Porto (IPO Porto)/Porto Comprehensive Cancer Center (Porto.CCC), 4200-072 Porto, Portugal; julio.oliveira@ipoporto.min-saude.pt (J.O.); pedro.reis.cruz@ipoporto.min-saude.pt (P.C.); i2567@ipoporto.min-saude.pt (B.S.); 2 Clinical Research Unit, Research Center of IPO Porto (CI-IPOP)/RISE@CI-IPOP (Health Research Network), Portuguese Oncology Institute of Porto (IPO Porto)/Porto Comprehensive Cancer Center Raquel Seruca (Porto.CCC Raquel Seruca), R. Dr. António Bernardino de Almeida, 4200-072 Porto, Portugal; 3 Abel Salazar Institute for the Biomedical Sciences (ICBAS), University of Porto, 4050-513 Porto, Portugal; tania.dias@ipoporto.min-saude.pt; 4 Molecular Oncology and Viral Pathology Group, Research Center of IPO Porto (CI-IPOP) & RISE@CI-IPOP (Health Research Network), Portuguese Oncology Institute of Porto (IPO Porto)/Porto Comprehensive Cancer Center (Porto.CCC), 4200-072 Porto, Portugal; beatriz.almeida@ipoporto.min-saude.pt (B.A.); hugo.sousa@ipoporto.min-saude.pt (H.S.); francisca.carvalho.dias@ipoporto.min-saude.pt (F.D.); 5 Department of Oncohematology, Portuguese Institute of Porto (IPO Porto)/Porto Comprehensive Cancer Center (Porto.CCC), 4200-072 Porto, Portugal; msousapimenta@ipoporto.min-saude.pt; 6 Research Department, Portuguese League Against Cancer Northern Branch (LPCC-NRN), 4200-172 Porto, Portugal; 7 Laboratory Medicine, Clinical Pathology Department, Portuguese Institute of Porto (IPO Porto)/Porto Comprehensive Cancer Center (Porto.CCC), 4200-072 Porto, Portugal; 8 Department of Occupational Health, Portuguese Institute of Porto (IPO Porto)/Porto Comprehensive Cancer Center (Porto.CCC), 4200-072 Porto, Portugal; rui.costa@ipoporto.min-saude.pt (R.C.); carlos.ochoa.leite@ipoporto.min-saude.pt (C.O.); 9 Research Innovation and Development Institute (FP-I3ID), Faculty of Health Sciences of Fernando Pessoa University (UFP), 4249-004 Porto, Portugal

**Keywords:** SARS-CoV-2, COVID-19, precision medicine, vaccines, humoral immunity

## Abstract

Background: Cancer patients face a greater risk of complications and death after contracting the SARS-CoV-2 virus. Booster doses of the COVID-19 vaccine were suggested to provide additional protection. This study aimed to assess how cancer patients’ immune systems respond to the booster shots and categorize their responses. Methods: We analyzed 735 samples from 422 individuals, including patients followed at the Portuguese Oncology Institute of Porto (IPO-Porto). Three cohorts were recruited, and blood samples were collected 3- and 6-months post-booster dose: cohort 1 cancer patients (also collected before the booster); cohort 2 cancer patients; and cohort 3 (healthy individuals). Humoral immune response was evaluated by analyzing IgG levels against the SARS-CoV-2 Spike (S) protein. IgG levels against the SARS-CoV-2 Nucleocapsid(N) protein was also analyzed in order to address previous contact with the virus. Results: Among Cohort 1 patients with solid tumors, when compared to pre-boost, IgG S levels increased 3 months after the boost and remained high after 6 months. Patients with hematologic tumors demonstrated lower IgG S levels at both timepoints. Comparing the IgG S levels among hematological tumors, solid tumors, and healthy individuals in both timepoints we observed that the healthy individuals had the strongest IgG S response, followed by the solid, and, lastly, the hematologic tumors. Solid tumor patients undergoing chemotherapy had reduced IgG S levels, especially those on high febrile neutropenia risk regimens. Conclusions: In conclusion, cancer patients have a weaker immune response to the SARS-CoV-2 vaccine, especially those with hematological cancers. Chemotherapy and febrile neutropenia risk further reduce booster effectiveness. Further research is needed to optimize vaccine timing for cancer patients undergoing chemotherapy.

## 1. Introduction

Coronavirus disease (COVID-19) is an infectious disease caused by a highly contagious and severe acute respiratory syndrome coronavirus 2 (SARS-CoV-2) [1]. This infection spread across the world rapidly and was declared a global pandemic by the World Health Organization (WHO) on the 11th of March 2020 [2,3]. This pandemic was responsible for more than six million deaths worldwide, demonstrating its importance as a public health emergency [3]. Reported illnesses have ranged from very mild (including some with no reported symptoms) to severe, including illness resulting in death [4]. COVID-19 can be divided in three clinical phases. The first phase being the time of infection, sometimes progressing to the second phase of pulmonary involvement and a third phase of an inflammatory state [5]. The final deep inflammatory state, known as cytokine storm, is caused by an excessive activation of pro-inflammatory genes, such as NF-kB, STAT-3, IL-6, IL-8 and G-CSF22, that can trigger an uncontrolled immune response, leading to lung tissue damage and progress into multiorgan failure [6]. In severe cases, SARS-CoV-2 infection can cause pneumonia and the excessive inflammation can result in acute respiratory distress syndrome (ARDS), leading to organ damage, hyperinflammatory state and lymphocytopenia, a hallmark that appears as both signature and prognosis of disease severity outcome [7,8]. In addition to age and gender, other conditions are considered as risk factors for SARS-CoV-2 infection, such as: asthma, obesity, hypertension, diabetes mellitus, chronic obstructive pulmonary disease (COPD), chronic kidney disease, smoking, and diseases that induce an immunosuppression state such as cancer [9,10,11,12]. To date, some efforts have been made to assess the safety of COVID-19 vaccines in immunocompromised populations, particularly in the gastrointestinal field [13]. Moreover, cancer patients are more susceptible to infection compared to healthy people and non-cancer patients due to the systemic malignancy-related immunosuppressive state and to active disease-oriented treatments, such as surgery, chemotherapy, radiotherapy, targeted therapies, and immunotherapy [14,15]. Moreover, patients with lymphoid malignancy, especially those with diminished T-cell responses and after B cell–depleting therapies, are particularly susceptible to viral infection complications, since they are severely immunocompromised due to the harsh effect of anti-cancer therapy targeting the bone marrow cells [16]. Some studies have already reported a very low antibody response in non-Hodgkin lymphoma (NHL) and chronic lymphocytic leukemia (CLL) compared to other cancer types and healthy individuals [17,18]. Solid tumors also presented low antibody response when compared to healthy individuals, and lung cancer patients are among the most affected [19]. To mitigate the surge in COVID-19 cases and manage the pandemic, a global effort was made to develop vaccines and therapeutic options This led to the emergence of new vaccines against SARS-CoV-2 and new antibody therapy options [20]. To date, vaccination and antiviral drugs are the main therapeutic agents pursued by the biotechnological and pharmaceutical companies to eradicate this pandemic. This underscores the importance of understanding how different populations respond to these methods and determining if there are sub-populations that might benefit more from one treatment over the other or require a combination of both. Cancer patients are an important subpopulation to study due to their immunocompromised state which could lead to a weaker response to vaccination [21,22]. Cytokine storm and a sustained inflammatory state are commonly associated with immune cell depletion, being manifested in most immunocompromised individuals [23,24]. This strong immunosuppression can lead to a dysfunctional antiviral response to natural viral infection and induced by vaccination. In addition to that, there is still some uncertainty in terms of the efficacy of the SARS-CoV-2 vaccines, as well as the extent of the humoral and cellular immune responses and the impact of related side effects [25]. Despite cancer patients have the indication for vaccination, it is of paramount importance to characterize the subgroups of low and non-responders, which represent a relevant public health issue, due to the risk of these patients developing COVID-19 infection and subsequent health complications [15,26]. This constraint is being supported by the development of new anti-COVID-19 therapies that could be applied to the non-responders to prevent the development of aggressive forms of infection [27]. Hence, the aim of our study was the identification of cancer patients’ subgroups with the lowest levels ofIgG response, following the booster dose of the COVID-19 vaccine over time. The accurate identification of patient groups with the lowest level of protection following COVID-19 vaccination is important to better target resources and interventions for the most vulnerable populations and, ultimately, design more accurate interventions to manage and protect these patients.

## 2. Materials and Methods

### 2.1. Study Design

To study the immune response to COVID-19 vaccine boost in cancer patients under active treatment the humoral immunity was evaluated through the quantification of IgG levels against SARS-CoV-2 Spike (IgGS) and IgG levels against SARS-CoV-2 Nucleocapsid (IgGN). The quantification of IgGS levels allows for the assessment of immunity induced by vaccination, while the presence of IgGN is indicative of previous exposure to the SARS-CoV-2 virus. Therefore, both antibodies were quantified in cohort 1 in Timepoint −1, Timepoint 1 and Timepoint 2. Regarding to cohort 2 and 3, IgG levels were assessed in Timepoint 1 and Timepoint 2.

### 2.2. Study Population

**Figure 1:**
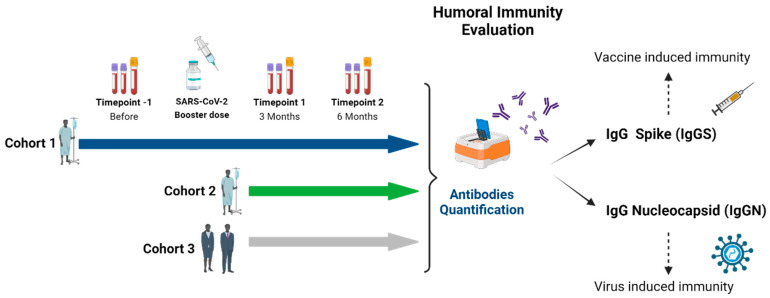
Study Design. Quantification of IgGS and IgGN in cohort 1 in Timepoint −1, Timepoint 1 and Timepoint 2. Regarding cohorts 2 and 3, IgG levels were assessed in Timepoint 1 and Timepoint 2.

This project was approved by the Portuguese Oncology Institute of Porto (IPO-Porto) ethics committee (CES IPO: 286/021) and all the patients’ enrolled in the study signed a written informed consent, in agreement with the Helsinki declaration principles. The inclusion criteria were patients age above 18 years, admitted at the IPO-Porto with hematological cancer or solid cancer under active treatment that were eligible for a booster dose of COVID-19 vaccination. The main vaccines administered to these patients were mRNA vaccines (Pfizer and Moderna). Therefore, this study included hematological and solid cancer patients that received SARS-CoV-2 vaccine-boosts: Cohort 1 with 56 patients collected samples in Timepoint −1, Timepoint 1 and Timepoint 2; Cohort 2 with 209 patients collected samples in Timepoint 1 and Timepoint 2; Cohort 3 with 138 healthy individuals collected samples in Timepoint 1 and Timepoint 2. As it is represented in Figure 1, up to three peripheral blood samples were collected from each patient. Regarding cohort 1, the first sample was collected before the patient received the booster dose of the vaccine and the other two samples at 3 and 6 months after the patient received the booster dose of the COVID-19 vaccine. Concerning the cohort 2 and 3, the samples were collected 3 and 6 months after the patient received the booster dose of the COVID-19 vaccine (Figure 1). For some recruited cancer patients, it was not possible to collect samples at all timepoints. The demographic and clinical data of cohort 1 and 2 are presented in Table 1. The healthy individuals recruited for cohort 3 (N = 138) consisted of 22 males (15.7%) and 118 females (84.3%) with mean ages of 54.9 and 53.5 years, respectively. The febrile neutropenia risk of the antineoplastic regimen at the time of the booster dose was classified as high if above 20%, medium if 10 to 20%, or low if below 10% [1]. Comorbities were classified according to their respective organ system.

### 2.3. Humoral Immunity Analysis

To study the immune response to SARS-CoV-2 booster vaccine in cancer patients’ humoral immunity was evaluated through the quantification of IgG levels against SARS-CoV-2 Spike (IgGS) and IgG levels against SARS-CoV-2 Nucleocapsid (IgGN). The quantification of the antibody’s levels to SARS-CoV-2 was performed using patients’serum samples. To detect specific anti-SARS-CoV-2 IgG antibodies, 200 μL of serum samples were examined using the Atellica^®^ IM SARS-CoV-2 IgG (sCOVG) immunoassay (Siemens Healthineers^™^) and EUROIMMUN ELISA kits for IgGN detection. Therefore, for cohort 1 IgG spike levels (U/mL) were compared between pre-boost group, 3- and 6-months post-boost group in hematological tumors and in solid tumors. Regarding to cohort 2, IgG spike levels between were compared between 3- and 6-months post-boost group in hematological tumors and solid tumours. Regarding cohort 3, which are made by healthy individuals, IgG spike levels were compared between 3- and 6-months post-boost group. For result interpretation, a non-reactive response was designated as anti-SARS-CoV-2 Spike and anti-SARS-CoV-2 Nucleocapsid IgG levels < 1.00, while a reactive response was labeled as SARS-CoV-2 Spike and SARS-CoV-2 Nucleocapsid IgG ≥ 1.00.

### 2.4. Statistical Analysis

The statistical analysis of the results was performed using SPSS software for Windows (Version 29.0) and GraphPad Prism (Version 8.0). After confirming normality parameters through Shapiro-Wilk and Kolmogorov-Smirnov tests, group differences were evaluated using Mann-Whitney *U* test.

## 3. Results

### 3.1. Quantification of IgG Levels Against SARS-CoV-2 Spike (S) and Nucleocapsid (N)

**Figure 2:**
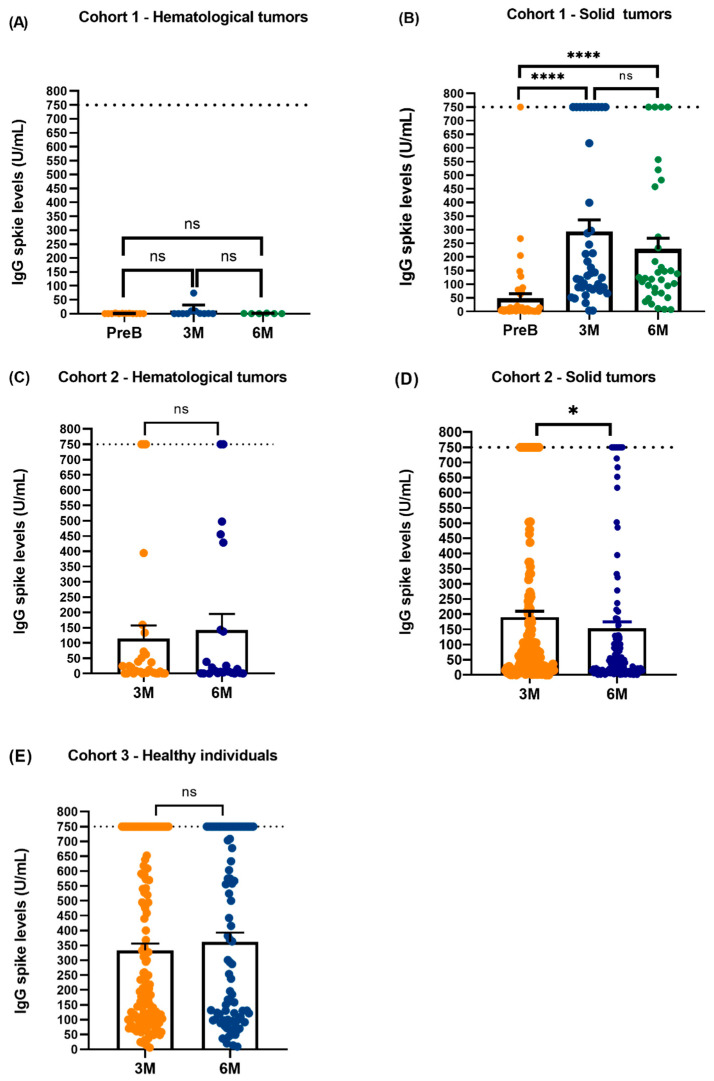
Quantification of IgG spike levels. Quantification of IgG spike levels. (**A**) IgG S levels between pre-boost (PreB) group (N = 12, 0.838 ± 0.702 U/mL), 3 months (3M) (N = 11, 8.933 ± 22.01 U/mL) and 6 months (6M) post-boost group (N = 6, 0.855 ± 0.666 U/mL) in hematological tumors; (**B**) IgG spike levels between PreB group (N = 44, 46.60 ± 122.9 U/mL) and 3M (N = 41, 291.5 ± 284.9 U/mL) and 6M (N = 34, 227.8 ± 240.2 U/mL) post-boost group in solid tumors. (**C**) IgG spike levels between 3M (N = 29, 114.2 ± 233.3 U/mL) and 6M group (N = 23, 143.2 ± 246.2 U/mL) in hematological tumors; (**D**) IgG spike levels between 3M (N = 180, 190.6 ± 257.9 U/mL) and 6M (N = 129, 153.8 ± 242.8 U/mL) post-boost group in solid tumors; (**E**) IgG S levels between 3M (N = 138, 332.9 ± 274.5 U/mL) and 6M (N = 88, 361.8 ± 291.6 U/mL) post-boost group in healthy individuals. Data presented as Mean ± SD, * *p* < 0,05, **** *p* < 0.0001. ns—not significant.

To study the immune response to SARS-CoV-2 booster vaccine in cancer patients the evaluation of the humoral immunity was performed through the quantification of IgGS and IgGN. Regarding cohort 1, IgGS levels were evaluated between pre-boost group, 3- and 6-months post-boost group in hematological tumors (n = 12) and in solid tumors (n = 44). Concerning to hematological tumors, any statistically significant difference was observed between the three timepoints and only 2 patients presented a slight increase on IgG levels 3 months after vaccination meaning that majority of these patients do not develop a IgG response after the booster dose of the vaccine (Figure 2A). Moreover, all hematological tumor patients were IgGN negative (−) in pre-boost, 3- and 6-months post-boost, which means that these patients did not have recent contact with the virus at the time of sample collection (Supplementary Table S1). In solid tumors the levels of IgG spike are higher in 3- and 6-months post-boost group comparing with pre-boost group, indicating that the humoral immunity of these patients increases after the booster dose of the vaccine (Figure 2B). Therefore, we can perceive that solid cancer patients had a better response to SARS-CoV-2 vaccination when compared to haematological cancer patients. Besides, in the pre-boost group 2 patients were IgGN (+) and 40 IgGN (−), in 3 months post-boost group, 11 patients were IgGN (+) and 28 were IgGN (−) and in 6 months post-boost group, 11 patients were IgGN (+) and 23 were IgGN (−) (Supplementary Table S1). Regarding cohort 2, no statistically significant differences were observed between 3 months and 6 months post-boost in IgGS levels in hematological tumors (n = 29) (Figure 2C). The number of patients IgGN (+) increased at 6 months post-boost since in 3 months post-boost group we observed that 4 patients were IgG NCP (+) and 23 IgG NCP (−) and in 6 months post-boost group, 7 patients were IgGN (+) and 16 were IgGN (−) (Supplementary Table S1). Regarding results from solid tumours (n = 180), as it is demonstrated at Figure 2D, IgGS levels decreased at 6 months post-boost. Moreover, the number of patients that had contact with the virus remain higher at 6 months with 20 patients were IgGN (+) and 154 IgGN (−) in 3 months post-boost group and 22 patients were IgGN (+) and 106 IgGN (−) in 6 months post-boost group (Supplementary Table S1). When we focus on cohort 3, which consists of healthy individuals (n = 138), all of them were IgGS positive in both timepoints (Figure 2E). We observed that 99 were IgGN (−) in 3 months post-boost group and 33 healthy individuals were IgGN (+) and in 6 months 48 were IgGN (−) in 3 months post-boost group and 38 individuals were IgGN (+) (Supplementary Table S1). Based on these preliminary results we can conclude that healthy individuals have a stronger immune response when compared to cancer patients.

**Figure 3:**
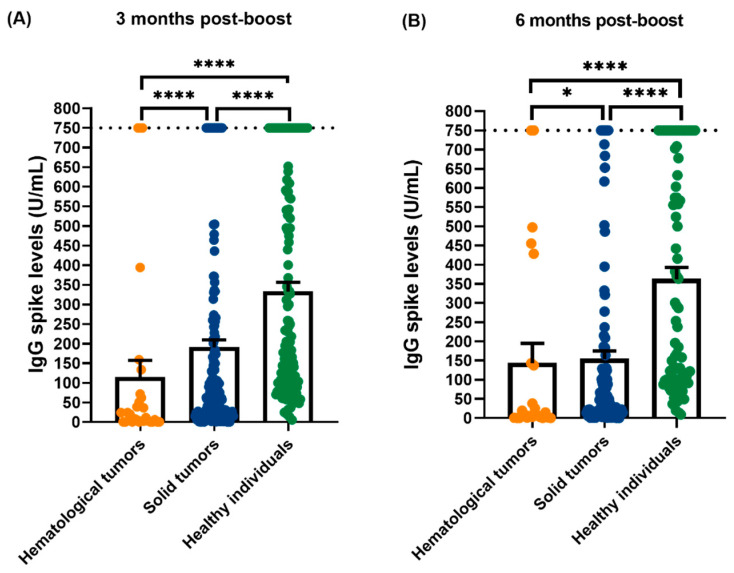
Quantification of IgGS levels in Timepoint 1 and Timepoint 2. (**A**) Quantification of IgG S levels in hematological tumors (N = 29, 114.2 ± 233.3 U/mL), solid tumors (N = 180, 190.6 ± 257.9 U/mL) and healthy individuals (N = 138, 332.9 ± 274.5 U/mL) in timepoint 1 (3 months after booster dose). (**B**) Quantification of IgG S levels in hematological tumors (N = 23, 143.2 ± 246.2 U/mL), solid tumors (N = 129, 153.8 ± 242.8 U/mL), and healthy individuals (N = 88, 361.76 ± 291.6 U/mL) in timepoint 2 (6 months after booster dose). Data presented as Mean ± SD, * *p* < 0.05, **** *p* < 0.0001.

Additionally, the IgG S levels between hematological tumors (N = 29), solid tumors (N = 180) and healthy individuals (N = 138) were compared in timepoint 1 (3 months after booster dose). As in Figure 3A, the antibodies levels are higher in healthy individuals (332.9 ± 274.5 U/mL) comparing with solid tumors patients (190.6 ± 257.9 U/mL) and with hematologic tumors (114.2 ± 233.3 U/mL). Also, patients with solid tumors, have higher IgG S levels comparing with hematological tumors. Relating to timepoint 2 (6 months after booster dose), we also compared the IgG S levels between hematological tumors (N = 23), solid tumors (N = 129) and healthy individuals (N = 88) and, as is demonstrated in Figure 3, the antibodies levels are higher in healthy individuals (361.76 ± 291.6 U/mL) comparing with solid tumors (153.8 ± 242.8 U/mL) and hematological tumors patients (143.2 ± 246.2 U/mL).

### 3.2. Stratified Analyzed of IgG Spike Levels in Hematological Patients

**Figure 4:**
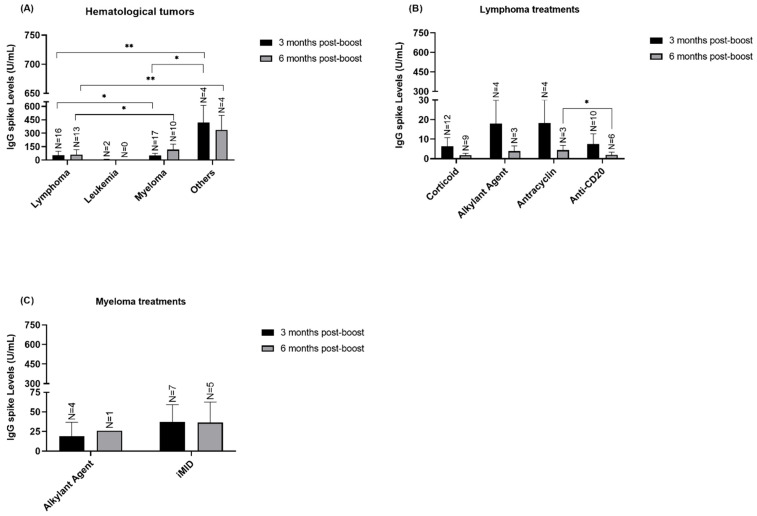
Quantification of IgG S according to hematological patients’ clinical variables -3- and -6-months post-boost in both cohort 1 and cohort 2. (**A**) Quantification of IgG S levels in lymphoma (median = 0.545 U/mL), leukemia (median = 5.215 U/mL), myeloma (median = 8.920 U/mL) and in other hematological tumor types (median = 441.8 U/mL) -3- and -6-months post-boost. (**B**) Quantification of IgG S levels according to corticoid, alkylant agent, antracyclin (median = 10.55 U/mL) and anti-CD20 (median = 0.49 U/mL) treatments in -3- and -6-months post-boost. (**C**) Quantification of IgG S levels according to alkylant agent and iMID treatment in -3- and -6-months post-boost. Data presented as Mean ± SEM, * *p* < 0.05, ** *p* < 0.01.

We proceeded to analyze the hematological patients IgG S titers according to the patients’ clinical characteristics (Figure 4). Regarding tumor type, it seems that myeloma patients presented highest levels of IgG S (Figure 4A) comparing to lymphoma patients in 3- and 6-months post-boost (*p* = 0.036 and *p* = 0.0197 respectively). Moreover, patients with other hematological malignancies as polycythemia vera and myelodysplastic syndrome demonstrated higher IgG S response than myeloma patients 3 months post-boost (*p* = 0.0165) and than lymphoma patients in 3- and 6-months post-boost (*p* = 0.0023 and *p* = 0.0034 respectively). Regarding the treatment schemes, when focusing on the lymphoma patients (Figure 4B) we observed that patients’ that had anti-CD20 seem to present lower IgG S response when compared with patient’s that had antracyclin agents in 6 months post-boost (*p* = 0.0476). Nevertheless, these results must be interpreted with caution due to the low N of samples.

### 3.3. Stratified Analyzed of IgG Spike Levels in to Solid Cancer Patients

**Figure 5:**
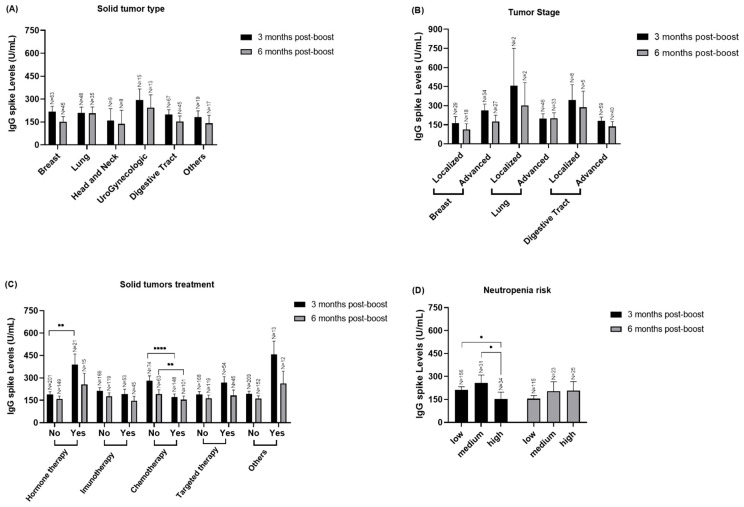
Quantification of IgG S according to solid cancer patients’ clinical variables -3- and -6-months post-boost in both cohort 1 and cohort 2. (**A**) Quantification of IgG S levels according to solid tumor types (**B**) Quantification of IgG S levels in patients with breast, lung and digestive tumors according to the AJCC 8th Edition tumor stage (I-III or IV). (**C**) Quantification of IgG S levels, in both timepoints, according to presence (median = 333.3 U/mL) and absence of hormone therapy (median = 79.89 U/mL) 3-months post-boost, presence (median = 63.23 U/mL) or absence of chemotherapy (median = 170.2 U/mL) 3-months post-boost, presence (median = 38.25 U/mL) or absence of chemotherapy (median = 94.35 U/mL) 6-months post-boost, of immunotherapy, of targeted therapy, and of and other therapies in patients with solid tumors. (**D**) Quantification of IgG S levels according to the low (median = 88.25 U/mL), medium (median = 101.30 U/mL) and high (median = 42.68 U/mL) febrile neutropenia risk in solid cancer patients -3- and -6-months post-boost (Data presented as Mean ± SEM, * *p* < 0.05, ** *p* < 0.01, **** *p* < 0.0001).

Regarding the solid tumor patients (Figure 5) we observed that patients that had chemotherapy presented a lower IgG response when compared to patients that did not had chemotherapy in both Timepoint 1 and Timepoint 2, *p* = 0.0004, *p* = 0.0065 respectively, (Figure 5C). However, 3 months post-boost, patients that had hormone therapy demonstrated higher levels of IgG S than patients that did not had, *p =* 0.0069 (Figure 5D). Moreover, we observed that patients with high febrile neutropenia risk also presented lower levels of IgG S when compared to patients that had medium (*p* = 0.0102) or low (*p* = 0.0368) risk (Figure 5D). We did not observe any statistically significant differences in terms of tumor type or tumor stage, but we must consider the low N of patients in some of the subgroups analyzed.

### 3.4. Stratified Analysis of IgG Spike Levels According to Solid and Hematological Patients’ Comorbidities and Clinical Variables

**Figure 6:**
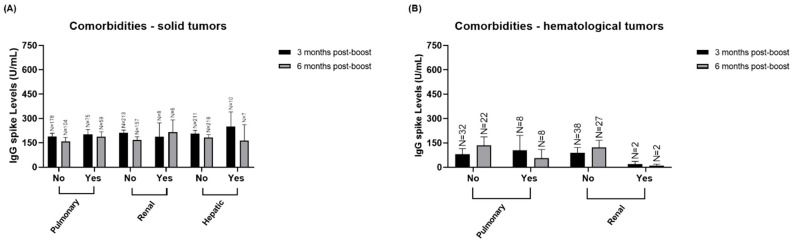
Quantification of IgG S according to pulmonary, renal and hepatic comorbidities. (**A**) Quantification of IgG S according to pulmonary, renal and hepatic comorbidities in solid tumor patients -3- and -6-months post-boost in both cohort 1 and cohort 2. (**B**) Quantification of IgG S according to pulmonary and renal comorbidities in hematological tumor patients -3- and -6-months post-boost in both cohort 1 and cohort 2. Data presented as Mean ± SEM.

We also analyzed the IgG S titers in solid and hematological patients according to various comorbidities and clinical variables. Figure 6 shows the analysis of IgG S levels in relation to pulmonary, renal, and hepatic comorbidities in patients with solid and hematological tumors at both -3- and -6-months post-boost. However, these comorbidities did not significantly impact IgG S levels.

**Figure 7:**
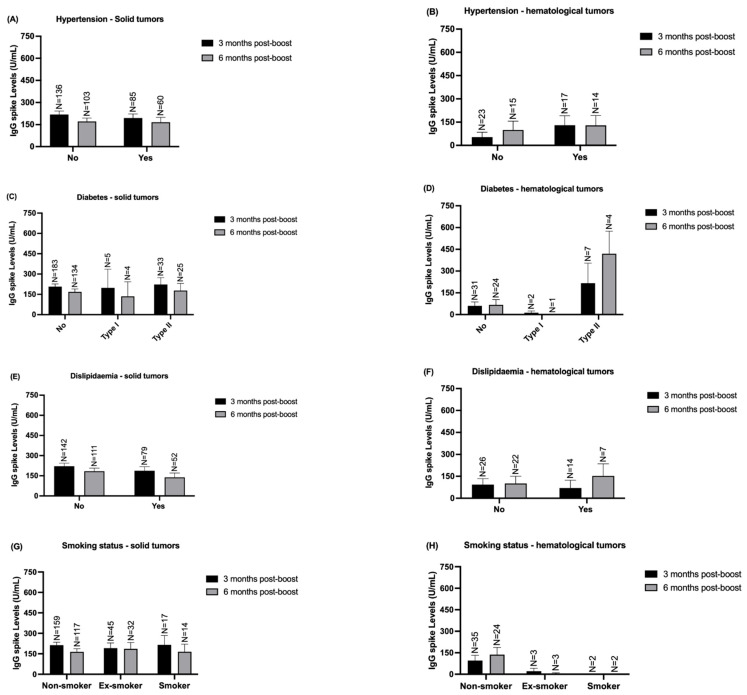
Quantification of IgG S according to solid and hematological patients’ comorbidities -3- and -6-months post-boost in both cohort 1 and cohort 2. (**A**) Quantification of IgG S levels according to hypertension in solid tumor patients in both timepoints. (**B**) Quantification of IgG S levels according to hypertension in hematological tumor patients in both timepoints. (**C**) Quantification of IgG S levels according to the presence or absence of diabetes in solid tumor patients -3- and -6-months post-boost. (**D**) Quantification of IgG S levels according to the presence or absence of diabetes in hematological tumor patients -3- and -6-months post-boost. (**E**) Quantification of IgG S levels based on the dyslipidemia in solid tumor patients in both timepoints. (**F**) Quantification of IgG S levels based on the dyslipidemia in hematological tumor patients in both timepoints. (**G**) Quantification of IgG S levels according to the smoking status of solid tumor patients -3- and -6-months post-boost. (**H**) Quantification of IgG S levels according to the smoking status of hematological tumor patients -3- and -6-months post-boost. Data presented as Mean ± SEM.

Additionally, we evaluated the influence of other comorbidities, such as hypertension, diabetes, dyslipidemia, and the smoking status of these patients, on the response to the COVID-19 vaccine booster (Figure 7). However, it appears that these clinical conditions do not affect IgG S levels production.

**Figure 8:**
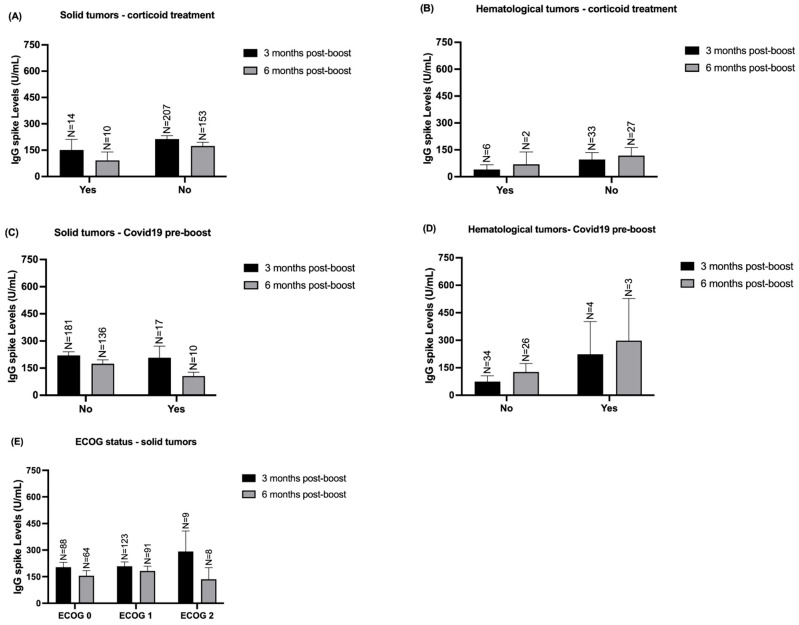
Quantification of IgG S according to solid and hematological patients’ clinical variables -3- and -6-months post-boost in both cohort 1 and cohort 2. (**A**) Quantification of IgG S levels according to the presence or absence of corticoid treatment in solid tumor patients -3- and -6-months post-boost. (**B**) Quantification of IgG S levels according to the presence or absence of corticoid treatment in hematological tumor patients -3- and -6-months post-boost. (**C**) Quantification of IgG S levels according to COVID-19 infection in solid tumor patients -3- and -6-months post-boost. (**D**) Quantification of IgG S levels according to COVID-19 infection in hematological tumor patients -3- and -6-months post-boost. (**E**) Quantification of IgG S levels according to the ECOG status in solid tumor patients in both timepoints. Data presented as Mean ± SEM.

Furthermore, we stratified our analysis based on the presence or absence of corticoid treatment, prior COVID-19 infection before the booster dose, and the ECOG status in solid tumor patients. None of these clinical variables affected IgG S levels, and consequently, the humoral immune response at both timepoints (Figure 8).

## 4. Discussion

The increase of knowledge on the real effectiveness of the immune responses after booster vaccines is of high priority in patients with solid tumors and in those with hematological malignancies. For cancer patients we may have a compromised immune system either due to the biology of cancer disease or because of the influence of the treatment that is being administrated to the patient. The immune response to the SARS-CoV-2 booster vaccine in cancer patients was studied by evaluating the humoral immunity. The study indicated that healthy individuals exhibited a more robust immune response to the SARS-CoV-2 booster vaccine compared to cancer patients. Within the cancer patient group, those with solid tumors responded better to the vaccine than those with hematological malignancies. Our results demonstrated that among the hematological patients, higher titers of antibodies were detected in patients with multiple myeloma. These findings may be due to the immune reconstitution induced by immunomodulatory drugs and/or alkylating agents. Lenalidomide (IMiD), for example, besides inducing degradation of the zinc finger proteins Ikaros and Ayolos, promotes the CD28 tyrosine phosphorylation in T cells, with downstream activation of NF-kB; whilst decreasing T regulatory and myeloid-derived suppressor cell counts. Regarding cyclophosphamide (an alkylating agent), the dose range varied accordingly to the disease and the immunochemotherapeutic protocol, hampering the detection of patterns. Indeed, non-Hodgkin lymphomas and B cell derived acute lymphoblastic leukemias are treated with higher doses of cyclophosphamide per comparison with multiple myelomas, where older patients may be enrolled in metronomic palliative cycling protocols empowering significantly lower doses of the agent. At lower doses, aldehyde-dehydrogenase depletion in T regulatory cells may induce an increase in the oxidative stress burst in these cells, inducing its death [3]. Without counter-regulation by regulatory lymphocytes, the germinal center reaction is favored, and humoral immunity more easily primed. Concerning myeloproliferative neoplasms, our patients were treated with hydroxycarbamide. Although known to partially reduce T cell counts, exposure to the drug does not significantly induce the expression of exhaustion markers in these cells. This corroborates the observation that although lower than in healthy individuals, there is a higher propensity to immunization by comparison with patients with acute leukemias or lymphoproliferative disorders (including chronic lymphocytic leukemia/small lymphocytic lymphoma). Among the different solid tumor types there were no significant differences in IgG S levels after the booster dose of the COVID-19 vaccination. A recent meta-analysis suggested no association between total serum IgG levels and solid cancer, and a lower IgG1/total IgG ratio has been similarly described in head and neck, gynecological, breast and colorectal cancer. While different immune response to vaccines have been described to depend on tumor type, it is possible that the different IgG S levels were not driven by the solid cancer type per se, but by other associated factors such as the corresponding treatment regimens [9]. Indeed, in our study, when analyzing the different treatment regimens, chemotherapy was associated with lower IgG S levels after the booster vaccine. Chemotherapy is myelosuppressive, which includes B cell cytotoxicity [10,11]. This treatment can induce long-term impairment of humoral immunity, with variable immune response to vaccines according to tumor type and immunosuppression [9,12,14,15,16,17,18,19]. To further analyse this, we evaluated the IgG S levels after the booster dose when under an antineoplastic treatment with high (>20%), medium (10 to 20%) or low (<10%) febrile neutropenia risk [1]. Although immunoglobulins are produced by plasma cells, neutropenia is considered a surrogate of the immune status, such that its resolution indicates immune reconstitution [20]. Thus, more myelosuppressive regimens, at the time of the booster dose, are associated with lower IgG S levels and could contribute to the different levels between tumour treatments, stages and types. In fact, in our study we observed that patients with high risk of neutropenia presented lower levels of IgG S when compared to patients that had medium or low risk. As for the higher levels detected in patients under hormone therapy, besides their lower number of cases, they were mostly under no chemotherapy or under low febrile neutropenia risk chemotherapy regimens. Thus, the patients who were not under hormone therapy could correspond to a selection of the patients under the higher febrile neutropenia risk chemotherapy regimens. Interestingly, the presence or absence of comorbities had no effect on the IgG levels, irrespective of their organ system. This finding, after the booster dose, is in line with previous findings of IgG levels after SARS-CoV-2 infection independent from comorbities [21]. Indeed, while comorbities have a significant impact in COVID-19 mortality and intensive care unit admission, their impact in antibody production is less clear [22]. Concerning tumor stage, we didn’t find different associations depending on cancer type since IgG S levels didn’t variate after the booster vaccine depending on advanced or localized stages. In the literature, IgG levels have been reported to not seem different between stages of breast cancer nor esophageal cancer, while in gastric cancer there are conflicting reports of no different and lower IgG levels between stage III or IV and stage I or II [23,24,25]. We also analyzed the IgG S titers in solid and hematological patients according to various comorbidities such as hypertension, diabetes, dyslipidaemia, and the smoking status of these patients. Additionally, we based our analysed according clinical variables as pulmonary, renal, and hepatic comorbidities. However, it appears that these clinical conditions do not affect IgG S levels production. Furthermore, presence or absence of corticoid treatment, prior COVID-19 infection before the booster dose, and the ECOG status in solid tumor patients didn’t impact IgG S levels, and consequently, the humoral immune response. Gathering all results from our study, we found that cancer patients had compromised immune system, leading to a weaker immune response to the SARS-CoV-2 vaccination. Despite the small number of patients with hematological tumors, this subgroup consistently showed low levels of IgG S following the booster dose of the COVID-19 vaccine over time. Therefore, this finding highlights the importance of increasing the sample size of patients with hematologic cancers, as this small number represents a limitation of the study. Unlike some clinical variables and comorbidities, the subgroup of cancer patients with higher risk of febrile neutropenia exhibited an inefficient humoral immune response compared to patients with medium or low risk. Moreover, our results suggest that chemotherapy significantly impacts the IgG response of cancer patients, impairing their ability to respond effectively to the COVID-19 vaccination. Therefore, it is crucial to evaluate the potential use of prognostic biomarkers to stratify vaccination response in cancer patients as well to further investigate the optimal timing for administering the vaccine to cancer patients undergoing chemotherapy [26,27,28,29,30,31,32]. Future studies should investigate the real biological meaning concerning the effectiveness in protection for cancer patients with solid tumors in comparison to those with haematological malignancies. Furthermore, under a precision medicine approach, the patient’s response profile, the complexity of each tumour patient and the dynamics of its disease must be considered for a closer look and also under the emergence of new SARS-CoV-2 strains and new improvements on the vaccines.

## Figures and Tables

**Table 1:** Clinical characteristics of patients with cancer included in this study in either cohort 1 or cohort 2.

		Gender	
		Male, n (%)	Female, n (%)
Patients, n (%)	284 (100%)	140 (49.3%)	144 (50.7%)
Patient Age (mean ± sd)	61.79 ± 11.16	63.67 ± 10.50	59.96 ± 11.48
SOLID TUMOR CASES, n (%)	240 (84.5%)	118 (49.2%)	122 (50.8%)
Tumor type, n (%)			
Breast, n (%)	68 (23.9%)	1 (1.5%)	67 (98.5%)
Lung, n (%)	51 (18.0%)	41 (80.4%)	10 (19.6%)
Head and Neck, n (%)	11 (3.9%)	10 (90.9%)	1 (9.1%)
Urogynecologic, n (%)	17 (6.0%)	11 (64.7%)	6 (35.3%)
Digestive Tract, n (%)	73 (25.7%)	46 (63.0%)	27 (37.0%)
Other, n (%)	20 (7.0%)	9 (45.0%)	11 (55.0%)
Cancer staging (AJCC 8th Edition)			
I–III, n (%)	76 (31.7%)	27 (35.5%)	49 (64.5%)
IV, n (%)	164 (68.3%)	91 (55.5%)	73 (44.5%)
Cancer Treatment			
ChemoT	160 (51.6%)	76 (47.5%)	84 (52,5%)
ImmunoT	58 (18.7%)	40 (69.0%)	18 (31.0%)
HormonoT	21 (6.8%)	7 (33.3%)	14 (66.7%)
TargetT	57 (18.4%)	22 (38.6%)	35 (61.4%)
Others	14 (4.5%)	1 (7.1%)	13 (92.9%)
Risk of Febril Neutropenia			
Low (<10%)	164 (68.3%)	98 (58.5%)	68 (41.5%)
Medium (10 to 20%)	35 (14.6%)	14 (40.0%)	21 (60.0%)
High (>20%)	39 (17.1%)	6 (19.5%)	33 (80.5%)
HEMATOLOGICAL MALIGNANCIES, n (%)	44 (15.5%)	22 (50.0%)	22 (50.0%)
Tumor type, n (%)			
Lymphoid	20 (7.0%)	9 (45.0%)	11 (55.0%)
Leukemia	2 (0.7%)	2 (100%)	0 (0%)
Myeloma	17 (6.0%)	10 (58.8%)	7 (41.2%)
Others	5 (1.8%)	1 (20.0%)	4 (80.0%)
Treatment			
Alkylating antineoplastic agent	16 (20.8%)	6 (37.5%)	10 (62.5%)
Anti-CD20 antibodies	19 (24.7%)	8 (42.1%)	11 (57.9%)
Anthracyclines	10 (13%)	3 (30.0%)	7 (70.0%)
Corticosteroids	22 (28.5%)	11 (50.0%)	11 (50.0%)
Immunomodulatory Drugs (IMiDs)	10 (13%)	7 (70.0%)	3 (30.0%)

## Data Availability

The data presented in this study are available on request from the corresponding author since these data are included in a larger ongoing project.

## Supplementary Materials

The following supporting information can be downloaded at: https://www.mdpi.com/article/10.3390/vaccines12111207/s1, Table S1: IgG N levels of the patients from cohort 1 and 2 in all the timepoints defined.
