# Supplementary material for: Humoral Response to SARS-CoV-2 Vaccine-Boost in Cancer Patients: A Case Series from a Southern European Cancer Center
Source: Vaccines (Basel). 2024 Oct 24;12(11):1207. doi: 10.3390/vaccines12111207 (PMC11598862; doi:10.3390/vaccines12111207)
Supplement: Supplementary file 1 [file vaccines-12-01207-s001.zip › vaccines-3267468-supplementary.pdf]

Supplementary Table 1 – IgG N levels of the patients from cohort 1 and 2 in all the timepoints defined.

Cohort 1			
**Hematological tumors**	**IgGN (+)**	**IgGN (-)**	**IgGN (Border)**
Pre-boost (N=12)	0	12	0
3 months post-boost (N=11)	0	11	0
6 months post-boost (N=6)	0	6	0
**Solid tumors**	**IgGN (+)**	**IgGN (-)**	**IgGN (Border)**
Pre-boost (N=44)	2	40	2
3 months post-boost (N=41)	11	28	2
6 months post-boost (N=34)	11	23	0
**Cohort 2**			
**Hematological tumors**	**IgGN (+)**	**IgGN (-)**	**IgGN (Border)**
3 months post-boost (N=29)	4	23	2
6 months post-boost (N=23)	7	16	0
**Solid tumors**	**IgGN (+)**	**IgGN (-)**	**IgGN (Border)**
3 months post-boost (N=180)	20	154	6
6 months post-boost (N=129)	22	106	1
**Cohort 3**			
**Healthy individuals**	**IgGN (+)**	**IgGN (-)**	**IgGN (Border)**
3 months post-boost (N=138)	33	99	8
6 months post-boost (N=88)	38	48	2
